# Dr. O. B. Webster

**Published:** 1914-10

**Authors:** 


### OBITUARY

---

Readers are requested to report promptly the death of all physicians in Western New York, or former residents of this region, or graduates of any medical school in Western New York, and to notify the families of the deceased of our desire to publish adequate obituary notices.

---

Dr. O. B. Webster, Buffalo, 18. ., for 50 years a physician of Lansing, Mich., died at Vassar, Mich., April 30, aged 87. Note.—No O. B. Webster is listed in the card index of the U. of B.

